# Complete Genome Sequence of Enterovirus D68 Detected in Classroom Air and on Environmental Surfaces

**DOI:** 10.1128/genomeA.00579-16

**Published:** 2016-06-16

**Authors:** John A. Lednicky, Tania S. Bonny, J. Glenn Morris, Julia C. Loeb

**Affiliations:** a Department of Environmental and Global Health, College of Public Health and Health Professions, University of Florida, Gainesville, Florida, USA; b Emerging Pathogens Institute, University of Florida, Gainesville, Florida, USA; c Department of Medicine, College of Medicine, University of Florida, Gainesville, Florida, USA

## Abstract

We amplified and sequenced the complete genome of enterovirus D68 (EV-D68) that had been collected from classroom air using a filter-based air sampling method and by swab sampling of environmental surfaces. Relatively high levels of EV-D68 genome equivalents were found per cubic meter of air by quantitative real-time reverse transcription-PCR (RT-PCR).

## GENOME ANNOUNCEMENT

Enterovirus D68 (EV-D68) (genus *Enterovirus*, family *Picornaviridae*) has reemerged globally as an important human respiratory pathogen (1–3). First identified in 1962 (4), respiratory diseases due to EV-D68 were rarely reported until the early 2000s (3, 5). In 2014, EV-D68 caused an outbreak in the United States that extended to early 2015 (3). During the recent U.S. outbreaks, EV-68 mostly affected children, causing clinical manifestations that ranged from mild respiratory illness to severe respiratory distress requiring hospitalization (3, 6). Alarmingly, sporadic cases of nonpolio paralysis/acute flaccid myelitis associated with residual limb weakness or other neurological deficits occurred during the recent American EV-D68 outbreaks (7–9). At least three EV-D68 clades exist (3, 6, 9); most recent outbreak strains in the United States, including those that caused acute flaccid myelitis, are from clade B1 (3, 6, 9). Relatively few EV-D68 genomes have been fully sequenced.

The virus described here was detected in 4 of 6 air sampler filters and 12 of 16 desktops of a classroom in a university, on 8 September 2015, a few weeks after fall season classes had started. To favor the detection of airborne virus, tests were performed immediately after the day’s last classroom session, before airborne virus would be removed in exhaust air by normal ventilation air exchanges. Active air sampling was performed at 9 liters/min for 1 h to sample 0.540 m^3^ of breathing air using a Sioutas Personal Cascade Impactor Sampler (PCIS) with polytetrafluoroethylene filters, as described previously (10), and desktop swab samples immersed in UTM viral transport medium (Copan Diagnostics, USA) (11). cDNA synthesis from viral nucleic acids extracted from filters (10) or swabs (11) was performed with avian myeloblastosis virus (AMV) reverse transcriptase and random hexamers, and PCR was performed using a panel of respiratory virus primers. Quantitative real-time reverse transcription-PCR (RT-PCR) tests (12) performed after the virus was identified indicated 400 to 5,000 genomic equivalents of EV-D68/m^3^ in the air samples. Viral RNA from the air sample with the highest concentration of virus was used for sequencing (13), and the complete viral genome was designated EV-D68/environment/Gainesville/1/2015. Phylogenetics indicate that the virus conforms to EV-D68 clade B1. Attempts to isolate the virus in cell cultures (13) from material extruded from filters or swab samples were unsuccessful due to rapid overgrowth of the cells by reovirus and/or adenovirus also present in the samples.

The EV-D68/environment/Gainesville/1/2015 genome has 3 nucleotide (nt) polymorphisms (C1817T, C3277A, and A4020G) that are present in the majority of EV-D68 strains of the 2014 U.S. outbreak (6), and in EV-D68/Haiti/1/2014 (GenBank accession no. KT266905.1) and EV-D68 MEX/DF/2014-InDRE2351 (GenBank accession no. KT825142.1). For these, the resulting amino acid substitutions T860N and S1108G at the cleavage sites of viral proteases P2A and P3C may affect their cleavage efficiency and lead to increased virus replication (6). As with our findings, high levels of airborne enteroviruses were detected in a pediatric clinic (14), and this may be a common finding in indoor settings with enterovirus-infected individuals. Our work also suggests that young adults can produce airborne EV-D68 and raises the question of whether airborne transmission is important for spreading the virus.

### Nucleotide sequence accession number.

The complete genome sequence of EV-D68/environment/Gainesville/1/2015 has been deposited in the GenBank database under the accession number KU509997.
